# Identification of CPT1A as a Prognostic Biomarker and Potential Therapeutic Target for Kidney Renal Clear Cell Carcinoma and Establishment of a Risk Signature of CPT1A-Related Genes

**DOI:** 10.1155/2020/9493256

**Published:** 2020-08-17

**Authors:** Yingkun Xu, Guangzhen Wu, Xue Ma, Jianyi Li, Ningke Ruan, Zhiyu Zhang, Yalei Cao, Yougen Chen, Qi Zhang, Qinghua Xia

**Affiliations:** ^1^Department of Urology, Shandong Provincial Hospital, Cheeloo College of Medicine, Shandong University, Jinan, Shandong 250021, China; ^2^Department of Urology, The First Affiliated Hospital of Dalian Medical University, Dalian, Liaoning 116011, China; ^3^Department of Pediatrics, Shandong Provincial Hospital, Cheeloo College of Medicine, Shandong University, Jinan, Shandong 250021, China; ^4^The Nursing College of Zhengzhou University, Zhengzhou, Henan 450001, China; ^5^Department of Urology, The Affiliated Yantai Yuhuangding Hospital of Qingdao University, Yantai, Shandong 264000, China; ^6^Department of Urology, Shandong Provincial Hospital Affiliated to Shandong First Medical University, Jinan, Shandong 250021, China

## Abstract

This study is aimed at investigating the expression, clinical significance, and biological role of CPT1A in kidney renal clear cell carcinoma (KIRC). We used the TCGA database and clinical pathology of tissue specimens to study the expression of CPT1A in KIRC. The expression of CPT1A in the kidney cancer tissue was significantly lower than that in the normal tissue. Survival curves demonstrated that the expression was correlated with prognosis in patients. We used the plasmid transfection method to explore the biological role of CPT1A in renal cancer cells and performed CCK-8, wound healing, and Transwell invasion experiments. The results demonstrated that CPT1A can inhibit the proliferation, migration, and invasion of renal cancer cells. Subsequently, we employed a bioinformatics analysis to further elucidate the role of CPT1A. The PPI network diagram was plotted, along with the coexpression diagram, between CPT1A and ten associated genes. The heat map was plotted, and the hazard ratio analysis of these eleven genes in KIRC was performed. Furthermore, the CPT1A, LPL, CPT2, and EHHADH genes were used to establish a reliable prognostic risk signature in KIRC. GSEA analysis demonstrated that CPT1A modulates tumor development via a variety of biological pathways in KIRC. We believe that CPT1A most likely suppresses tumor progression by employing tumor “slimming” in KIRC. Collectively, the results indicate the potential of CPT1A as a novel prognostic indicator and potential therapeutic target in KIRC.

## 1. Introduction

Renal cell carcinoma (RCC) is a malignant tumor originating from the renal parenchymal urothelial system. It is the second most common tumor occurring in the urinary system, accounting for about 85% of renal malignancies worldwide [1]. More than 70,000 new cases of RCC are reported globally each year [2]. RCC can be divided into different subtypes based on different biological characteristics, including kidney renal clear cell carcinoma (KIRC), kidney renal papillary cell carcinoma (KIRP), and kidney chromophobe (KICH). KIRC is the most common fatal cancer of the adult kidney, accounting for 70% to 80% of reported cases of RCC. It does not respond to radiochemotherapy and exhibits a high risk of metastasis [3, 4]. Despite the progress made in the diagnosis and treatment of KIRC in recent years, surgical treatment remains the primary form of therapy. Approximately 20-40% of patients exhibit postoperative metastasis or local recurrence, which is accompanied by a poor prognosis [5]. Therefore, there is an urgent need for identifying biomarkers for predicting prognosis and therapeutic effectiveness.

Previous studies have demonstrated that metabolic reprogramming is one of the most prominent characteristics of tumor cells and is primarily reflected in the abnormal metabolism of glucose, fatty acids, and amino acids [6–9]. Compared with those on glucose and amino acid metabolism, relatively few studies have been conducted on abnormal fatty acid metabolism in tumor cells. However, many recent studies have confirmed a significant correlation between abnormal fatty acid metabolism and tumor development [10, 11]. In contrast, phospholipids and cholesterol can affect tumor cell proliferation by directly participating in the composition of the cell membrane. Lipids and their intermediate metabolites are important active molecules in the physiological processes of cells [12]. Abnormal fatty acid metabolism is very important in renal clear cell carcinoma [10]. Thus, investigation of the abnormal fat metabolism in renal clear cell carcinoma cells can be of high theoretical and practical value.

CPT1 has three different subtypes, namely, CPT1A, CPT1B, and CPT1C. The genetic sequences of CPT1A and CPT1B exhibit high similarity; each of these subtypes consists of 18 coding and two noncoding exons and exhibits 63% similarity with the other with respect to amino acid composition. However, the difference in cellular dynamics between the two sequences determines their different functions and tissue distributions. Compared with CPT1B, CPT1A has a higher affinity for carnitine, and the expression of CPT1A can be regulated in a variety of ways, such as transcription factor regulation, miRNA regulation, hormone regulation, and metabolite feedback regulation [13–15]. Therefore, CPT1A regulates the oxidation of fatty acids in the body to a higher degree and affects the routine activities of the body, thus playing an extremely important role.

Renal clear cell carcinoma tumors are transparent because of the large amount of lipid deposition inside the cells. Changes in VHL tumor suppressors that stabilize hypoxia-inducible factors (HIFs) are the most common molecular feature of clear cell tumors. The HIF gene can inhibit tumor progression by regulating the lipids deposited in renal clear cell carcinoma cells. CPT1A is a direct target of HIF. CPT1A demonstrated significantly lower expression in renal clear cell carcinoma tissue than in normal renal tissue [16]. However, research on CPT1A in renal clear cell carcinoma is still lacking. Therefore, our research focused on exploring the expression and clinical significance of CPT1A in renal clear cell carcinoma, studying the biological effects of CPT1A on renal clear cell carcinoma cells, and using a number of biological methods to explore the mechanism underlying these effects. Finally, four genes, including CPT1A, were used to establish a prognostic risk signature in patients with renal clear cell carcinoma.

## 2. Materials and Methods

### 2.1. KIRC Tissue Samples

From January 2013 to January 2018, renal cancer tissues and paired normal tissues adjacent to cancer (cut margins > 2 cm from the tumor) were excised from 50 patients in our hospital who underwent surgery and no other treatments; all of these were verified by a pathologist. This study was approved by the Medical Ethics Committee of our hospital, and the patients provided informed consent. The pathological staging of renal cancer was determined following the American Cancer Society (AJCC) 7th Edition 2010 Renal Cancer Staging Standard. After the specimen was excised, the sample was cut into small pieces, followed by the addition of an RNA protection agent. The samples were then placed in a refrigerator at -80°C.

### 2.2. Immunohistochemical Assay

KIRC specimens were fixed with 4% paraformaldehyde and embedded in paraffin for sectioning. Citrate buffer (pH 6.0, 5 min) was used for antigen recovery. For labeling, the sections were incubated with an anti-CPT1A antibody at 4°C overnight. The sections were incubated with secondary antibodies for 30 min and tested with a DAB kit (Abcam), and the nuclei were stained with hematoxylin (Abcam) according to the manufacturer's protocol. The results were evaluated by two independent pathologists.

### 2.3. Cell Culture and Antibodies

The 786-O and ACHN cell lines were purchased from the Cell Bank of the Chinese Academy of Sciences. All cells were cultured in an atmosphere containing 5% CO_2_ in the presence of penicillin and streptomycin at 37°C. Anti-CPT1A antibody (Abcam, 128568) and anti-*β*-actin antibody (Abcam, 8227) were used for the experiments.

### 2.4. Establishment of a Renal Cell Line Overexpressing CPT1A

We inoculated 2 × 10^5^ 786-O and ACHN cells collected in the logarithmic growth phase into 6-well plates. The plasmid was transfected the next day. The method was as follows. First, Lipofectamine 2000 (Invitrogen) and the plasmid fragments were diluted with a serum-free medium. The dilutions were allowed to stand for 5 min, followed by mixing. They were then allowed to stand for another 30 min. Using a pipette, 100 *μ*L of the above mixture was added to the cells in a 6-well plate. After 6 h of incubation, the medium containing serum was changed to continue the culture until 24 h. Following digestion with trypsin and subsequent collection of cells, they were used for qRT-PCR, western blotting, proliferation, migration, and invasion experiments.

### 2.5. RNA Extraction and qRT-PCR

Cellular RNA was extracted using a commercial kit (OMEGA Company, catalog number R6834). Subsequently, the extracted RNA was used to synthesize cDNA using a reverse transcription kit (Takara Corporation, catalog number RR047A). The sequences of CPT1A primers used for PCR were F-5′-CTGGACAATACCTCGGAGCC-3′ and R-5′-AACGTCACAAAGAACGCTGC-3′. Finally, a relative quantitative method was used to calculate the relative expression level in each group.

### 2.6. Western Blotting

Cell protein was loaded at 30 *μ*g per well in a 10% polyacrylamide gel (SDS-PAGE) and then separated at 100 V and transferred to a PVDF membrane using 200 mA constant current. Primary antibody was added after blocking the membrane with 5% skimmed milk powder for 1 h. The cells were incubated overnight at 4°C, rinsed thoroughly with TBST (10 min × 3 times) followed by the addition of secondary antibody and incubation for 1 h. The cells were again rinsed thoroughly with TBST, and color was developed using an ECL color reagent kit.

### 2.7. Cell Proliferation Assay

The 786-O and ACHN cells were seeded at 200 *μ*L/well (approximately 5 × 10^3^ cells) in 96-well plates 48 h after transfection and were placed in an incubator containing 5% CO_2_ at 37°C to continue the culturing process. After 1, 2, 3, 4, and 5 days, 110 *μ*L of a mixture of Cell Counting Kit-8 (CCK-8) and serum-free culture solution (1 : 10) was added to each well according to the CCK-8 manufacturer's instructions. After incubation for 1 h in the dark, the optical density of each well was measured at 450 nm in a microplate reader.

### 2.8. Wound Healing Assay

CPT1A was overexpressed in 786-O and ACHN cells. When the cells in the 6-well plate reached approximately 90% confluence, a pipette tip was used to create a wound in the cell layer in each group. The shed cells were gently rinsed with PBS, and a serum-free medium was added. A microscope was used for observation and to capture images immediately and 24 h after the scribing process. Finally, the relative migration ability of each group of cells was analyzed.

### 2.9. Transwell Assay

It is necessary to apply a Matrigel coating to the Transwell chamber. The addition of 500 *μ*L of a serum-containing medium to the bottom of the chamber was performed followed by the addition of 50 *μ*L of a serum-free medium to the upper chamber. CPT1A-overexpressing cells were then added to the chamber at a density of 5 × 10^4^ cells/chamber and cultured for 48 h. The cells were then fixed with 4% paraformaldehyde for 15 min, stained with crystal violet for 20 min, washed thrice with PBS, and placed in a fume hood to dry; finally, cells that invaded to the bottom of the chamber were observed and photographed under a microscope.

### 2.10. Bioinformatics Analysis

First, we downloaded the mRNA and clinical data of the KIRC dataset from the TCGA database. The R language drawing software package was used to derive a scatterplot of CPT1A expression and a box plot illustrating the association between the expression and clinical features. We identified ten genes with the strongest correlation with CPT1A through the String website and generated a PPI map for the eleven genes. We used the corrplot software package for coexpression analysis, the pheatmap software package to generate heat maps, and the survival software package to analyze and obtain survival curves. Subsequently, the LASSO regression curve was used to establish a risk signature in KIRC and obtain survival curves for high- and low-risk groups. To verify the accuracy of this model, we used the survivalROC software package to analyze and draw an ROC curve. Based on this risk model, we conducted univariate and multivariate Cox analyses. Finally, we conducted a GSEA analysis for CPT1A in KIRC to explore the biological pathways of gene enrichment.

### 2.11. Statistical Analysis

In this study, the Prism software (GraphPad, CA, USA) was used for statistical analysis of the data. Statistical significance of results of comparison between the two groups was evaluated using the two-tailed *t*-test and one-way analysis of variance. A *P* value of <0.05 was considered statistically significant.

## 3. Results

### 3.1. The Expression of CPT1A in KIRC Is Downregulated and Associated with Poor Prognosis of Patients

**Figure 1:**
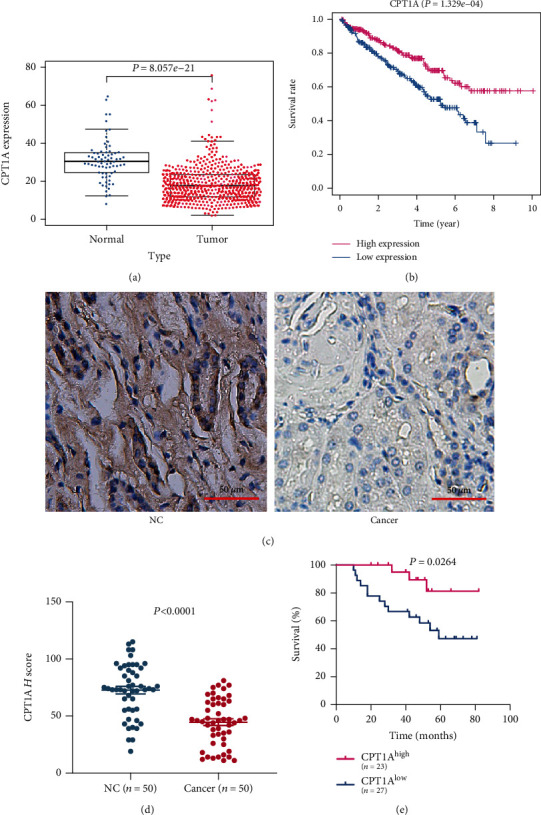
CPT1A is downregulated in KIRC and is associated with the overall survival rate of KIRC patients. (a) The scatterplot of CPT1A expression in the KIRC dataset of the TCGA database. (b) The survival curve of CPT1A obtained using the KIRC dataset of the TCGA database. (c) Typical immunohistochemical images of CPT1A in kidney cancer tissues and normal kidney tissues. (d) Scatterplot of the *H* scores of CPT1A expression in 50 cancerous and normal kidney tissues. (e) Survival curve obtained based on the clinical data of kidney cancer patients collected by us.

To evaluate the expression of CPT1A in KIRC, we used the TCGA database and clinical-pathological tissue examination. Using data from the TCGA database, we observed that the expression of CPT1A in renal clear cell carcinoma tissue was significantly lower than that in normal kidney tissue (*P* = 8.057*e* − 21) (Figure 1(a)). The immunohistochemical images of pathological sections also demonstrated the same results (*P* < 0.0001) (Figures 1(c) and 1(d)). Similarly, we used a combination of data from the TCGA database and clinical data for evaluating the correlation between the expression of CPT1A and the prognosis of patients in kidney cancer. In the TCGA database, we observed that the overall survival rate of RCC patients with low expression of CPT1A was significantly worse than that of RCC patients with high expression of CPT1A (*P* = 1.329*e* − 04) (Figure 1(b)). Similar results were observed upon evaluating a survival curve constructed based on patient follow-up data. The results imply that the lower the expression of CPT1A, the worse is the prognosis (*P* = 0.0264) (Figure 1(e)).

### 3.2. Low Expression of CPT1A Is Associated with Multiple Clinicopathological Features

**Figure 2:**
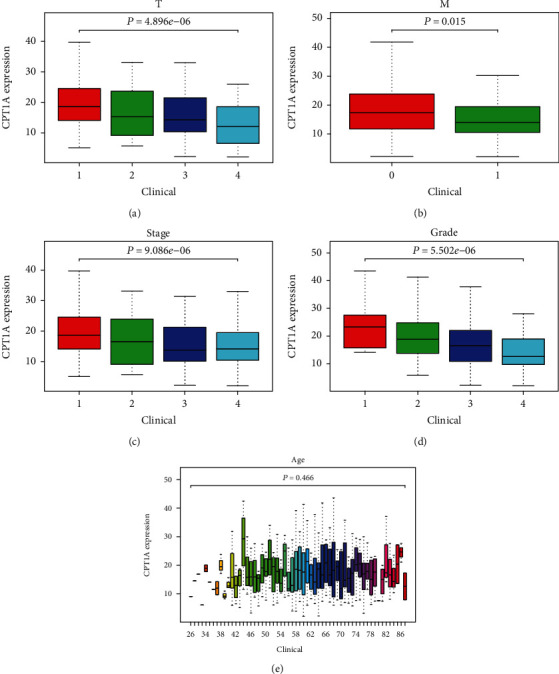
The relationship between the expression of CPT1A and various clinicopathological parameters in KIRC patients. (a) Tumor. (b) Metastasis. (c) Stage. (d) Grade. (e) Age.

Correlation between the expression of CPT1A and various clinicopathological characteristics as well as the patient age and gender was evaluated. We observed that the expression of CPT1A was associated with the tumor size (*P* = 0.035) and Fuhrman grade (*P* = 0.019) of patients using the clinical data obtained by us (Table 1). Subsequently, an in-depth analysis of the clinical data from TCGA demonstrated that the expression of CPT1A was associated with tumor (*P* = 4.896*e* − 06), metastasis (*P* = 0.015), stage (*P* = 9.086*e* − 06), and grade (*P* = 5.502*e* − 06) (Figures 2(a)–2(d)). In conclusion, the results demonstrated that the pathological subtype worsens with the decrease in the expression of CPT1A in RCC patients. However, no correlation was observed between CPT1A expression and age (Figure 2(e)).

### 3.3. CPT1A Can Inhibit the In Vitro Proliferation of Renal Clear Cell Carcinoma Cells

**Figure 3:**
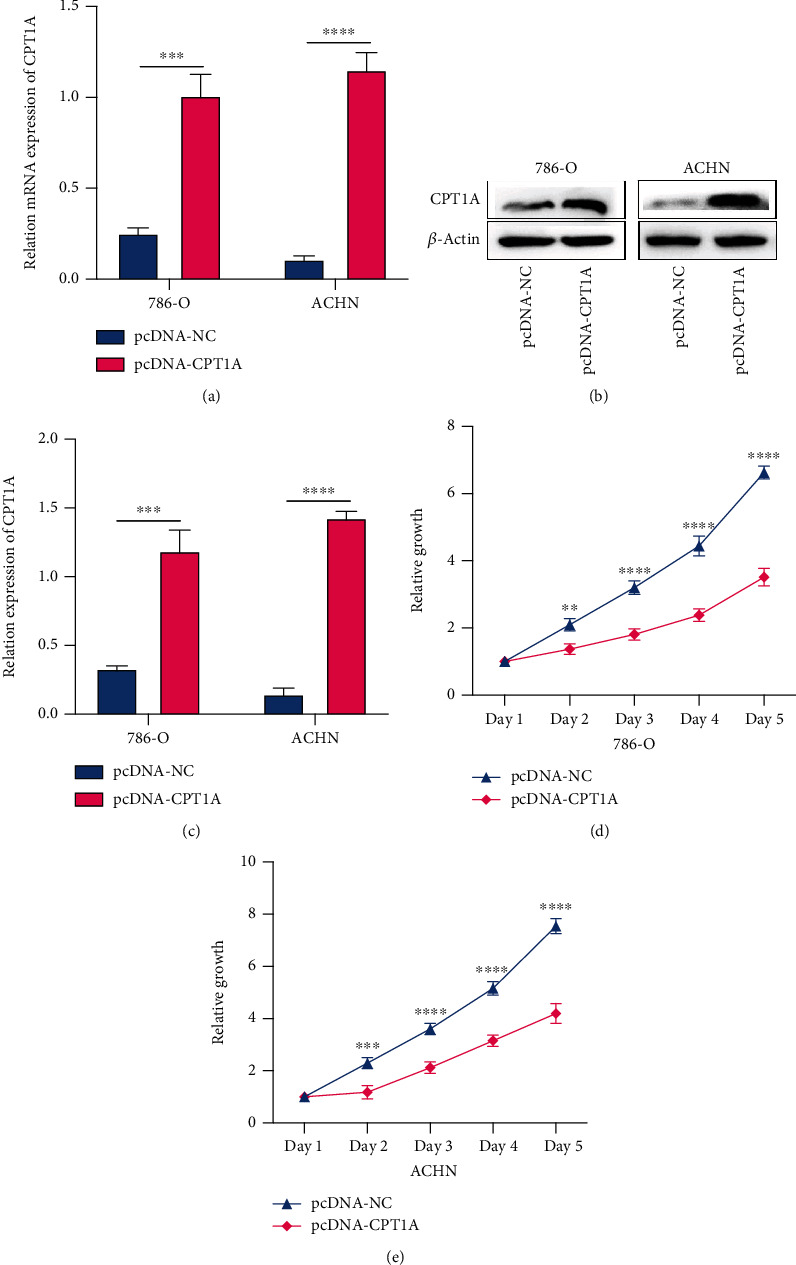
Verification of CPT1A overexpression and cell proliferation experiments. (a) Histogram of qRT-PCR results after plasmid transfection. (b, c) Image and histogram of western blotting results after plasmid transfection. (d, e) The results of CCK-8 experiments in the two cell lines 786-O and ACHN following plasmid transfection. ^∗∗^*P* < 0.01; ^∗∗∗^*P* < 0.001; ^∗∗∗∗^*P* < 0.0001.

To evaluate the potential role of CPT1A in KIRC, a kidney cancer cell line overexpressing CPT1A was established. We used the plasmid transfection method to overexpress CPT1A in two cell lines, namely, 786-O and ACHN. Subsequently, qRT-PCR and western blotting were used to verify the success of transfection. We observed that both the mRNA and protein expression in the CPT1A overexpression group were significantly higher than those in the control group (Figures 3(a)–3(c)). We performed the CCK-8 assay to determine whether CPT1A can affect the proliferation of kidney cancer cells. The proliferation of cells in the CPT1A overexpression group was significantly inhibited (Figures 3(d) and 3(e)) in both 786-O and ACHN cell lines.

### 3.4. CPT1A Can Inhibit the Migration and Invasion Abilities of Renal Clear Cell Carcinoma Cells In Vitro

**Figure 4:**
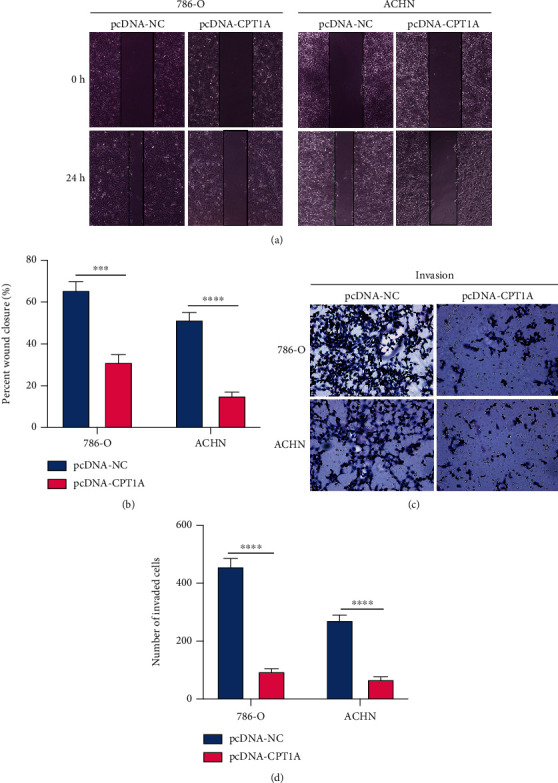
Wound healing and cell invasion experiment. (a, b) Images from the wound healing experiments in 786-O and ACHN cell lines after plasmid transfection and the histogram obtained upon the analysis of the results. (c, d) Images of the Transwell invasion experiment of the two cell lines and the histogram obtained upon the analysis of the results. ^∗∗∗^*P* < 0.001; ^∗∗∗∗^*P* < 0.0001.

To investigate whether CPT1A affects the migration and invasion abilities of renal clear cell carcinoma cells, we performed wound healing and Transwell invasion experiments in 786-O and ACHN cell lines. The wound healing experiment demonstrated that the wound healing rates of the 786-O and ACHN cells overexpressing CPT1A were significantly lower than those of the control group (Figure 4(a)), and a histogram was drawn to show the difference (Figure 4(b)). The numbers of invasive 786-O and ACHN cells overexpressing CPT1A were significantly reduced compared to that in the control group, as observed using the Transwell invasion experiment (Figure 4(c)). This result was statistically analyzed, and a histogram was obtained, as shown in Figure 4(d).

### 3.5. Coexpression Analysis of CPT1A and Ten Associated Genes, as Well as Gene Expression and Hazard Ratio Analysis in KIRC

**Figure 5:**
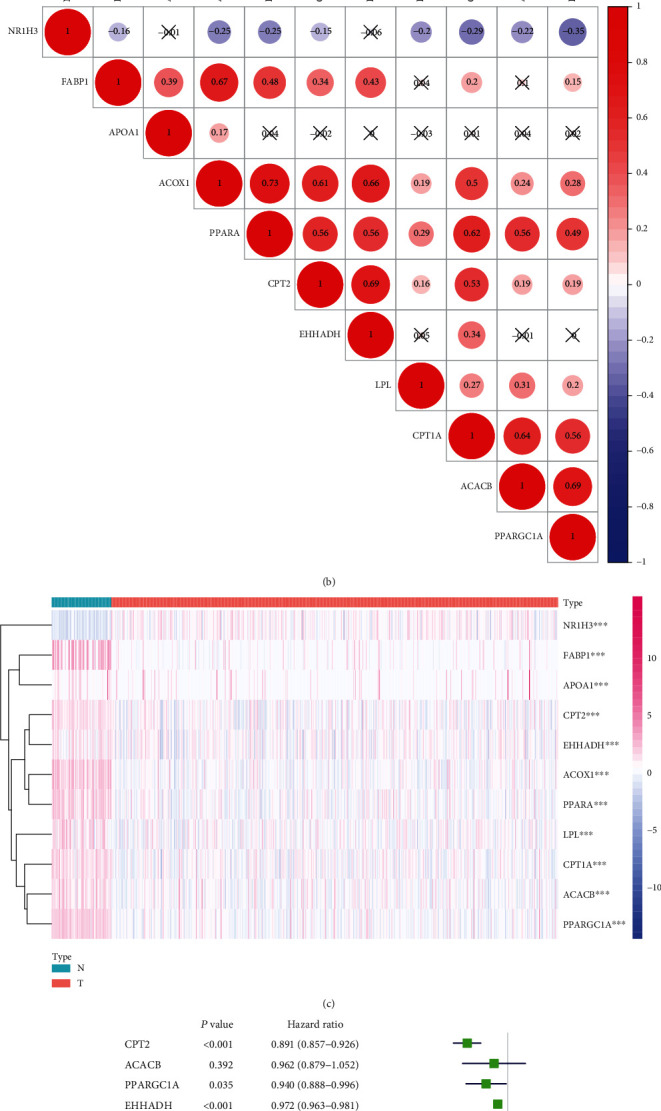
Coexpression analysis of CPT1A and ten associated genes along with the gene expression and hazard ratio analysis in KIRC. (a) PPI network diagram between CPT1A and the ten most relevant genes. (b) Schematic diagram of the coexpression of these eleven genes. (c) Heat map demonstrating the expression of the eleven genes in KIRC. The higher the intensity of red color, the higher is the expression. The higher the intensity of blue color, the lower is the expression. (d) Hazard ratio analysis of the 11 genes in KIRC. ^∗∗∗^*P* < 0.001.

To explore the biological functions of CPT1A and associated genes, ten most relevant genes associated with CPT1A were identified from the PPI network constructed in the String website. A strong interaction was observed in the network between the eleven genes (Figure 5(a)). We subsequently used the R language to evaluate the coexpression of the eleven genes (Figure 5(b)). A positive correlation was observed between CPT1A and PPARA expression. To display the relative expression of these eleven genes in KIRC, we used a software package of the R language to draw a heat map (Figure 5(c)). The expression of the eleven genes was significantly different between kidney cancer tissue and normal kidney tissue. CPT2, PPARGC1A, EHHADH, CPT1A, ACOX1, PPARA, and LPL exhibit protective effects in KIRC, as observed through hazard ratio analysis (Figure 5(d)).

### 3.6. Establishment of a Risk Signature in KIRC

**Figure 6:**
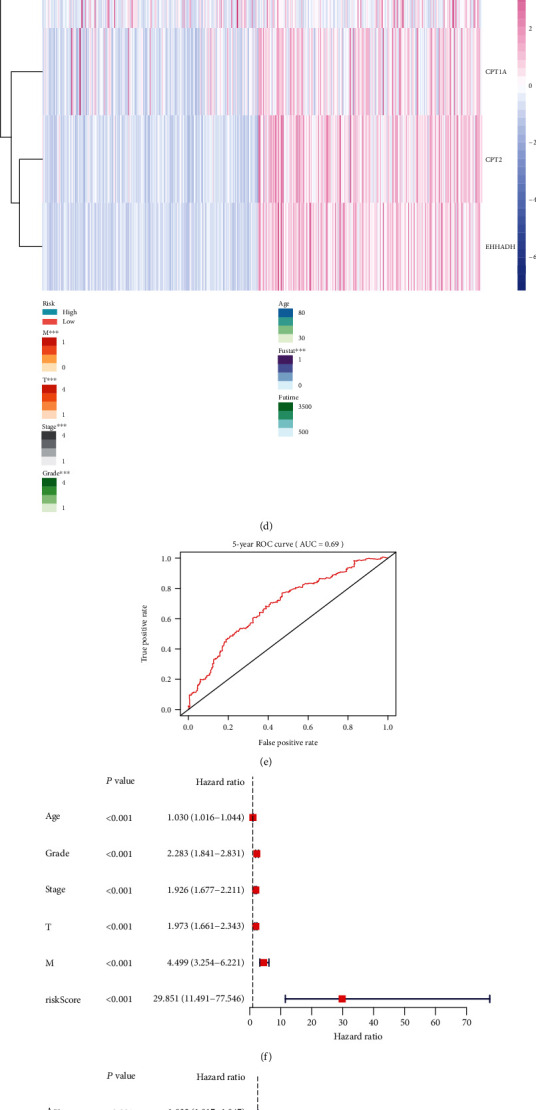
The process of establishing a risk signature. (a, b) Four genes were selected by LASSO Cox regression analysis. (c) The survival curve obtained based on this model. Red corresponds to the high-risk group, and blue corresponds to the low-risk group. (d) Based on the risk signature associated with prognosis, a heat map using data regarding the clinicopathological features of the patient was drawn. The higher the intensity of the red color, the higher is the expression. The higher is the intensity of the blue color, the lower is the expression. (e) Five-year ROC curve. (f) Univariate Cox analysis. (g) Multivariate Cox analysis. ^∗∗∗^*P* < 0.001.

We built a model in KIRC by drawing a LASSO regression curve. This model included four genes, CPT1A, CPT2, EHHADH, and LPL (Figures 6(a) and 6(b)). Upon dividing KIRC patients into high-risk and low-risk groups based on the model, the prognosis of the high-risk group was observed to be significantly worse than that of the low-risk group (*P* = 3.933*e* − 09) (Figure 6(c)). We also observed a remarkable correlation between the high- and low-risk groups based on this model, along with metastasis, tumor, stage, grade, and fustat of patients (Figure 6(d)). The AUC value of the five-year ROC curve was 0.69, indicating a good prediction accuracy of the model (Figure 6(e)). In the subsequent univariate Cox analysis, age, grade, stage, tumor, metastasis (Figure 6(f)), and risk score were identified as the risk factors. In multivariate Cox analysis, age, grade, stage, and risk score were identified as independent risk factors (Figure 6(g)).

### 3.7. CPT1A May Be Involved in Biological Pathways Associated with the Occurrence and Development of KIRC

**Figure 7:**
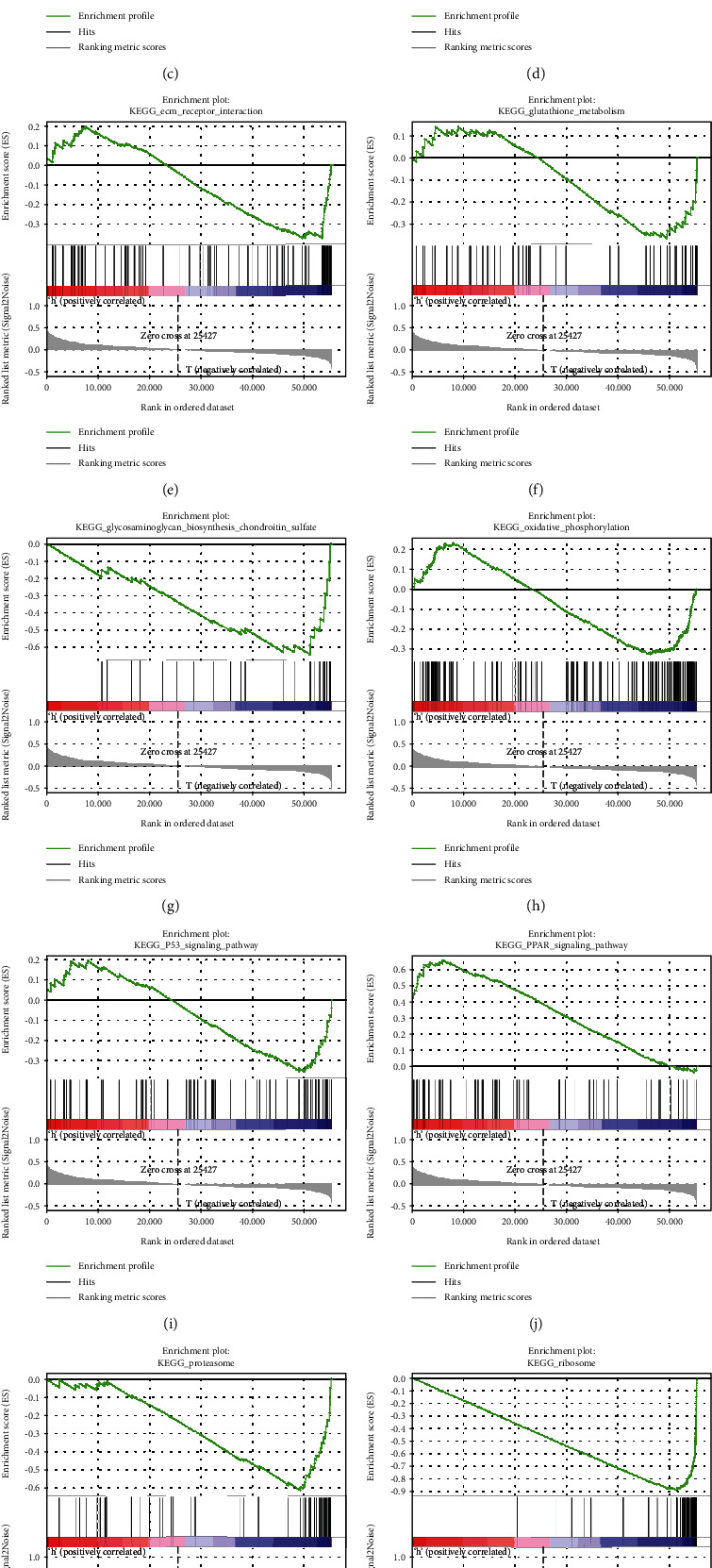
GSEA analysis for CPT1A in KIRC. (a) Adipocytokine signaling pathway. (b) Citrate cycle/TCA cycle. (c) Complement and coagulation cascades. (d) Cytokine-cytokine receptor interaction. (e) ECM receptor interaction. (f) Glutathione metabolism. (g) Glycosaminoglycan biosynthesis chondroitin sulfate. (h) Oxidative phosphorylation. (i) P53 signaling pathway. (j) PPAR signaling pathway. (k) Proteasome. (l) Ribosome. (m) Systemic lupus erythematosus.

To explore the KEGG pathways associated with the CPT1A gene in KIRC, we conducted a GSEA analysis. We observed that the adipocytokine signaling pathway (Figure 7(a)), citrate cycle/TCA cycle (Figure 7(b)), and PPAR signaling pathway (Figure 7(j)) demonstrated a positive correlation with CPT1A expression. The complement and coagulation cascades (Figure 7(c)), cytokine-cytokine receptor interaction (Figure 7(d)), ECM receptor interaction (Figure 7(e)), glutathione metabolism (Figure 7(f)), glycosaminoglycan biosynthesis chondroitin sulfate (Figure 7(g)), oxidative phosphorylation (Figure 7(h)), P53 signaling pathway (Figure 7(i)), proteasome (Figure 7(k)), ribosome (Figure 7(l)), and systemic lupus erythematosus (Figure 7(m)) pathways were negatively correlated with CPT1A expression.

## 4. Discussion

CPT1A is a key enzyme that regulates the oxidation of fatty acids in cells, primarily located in the inner mitochondrial membranes. It is responsible for transporting free fatty acids from the cytoplasm to the mitochondria, allowing fatty acids to enter the oxidation process [17, 18]. CPT1A can play a very important role in fatty acid metabolism. In recent years, extensive oncology research involving this enzyme has been conducted [19]. Studies have demonstrated that CPT1A is highly expressed in ovarian cancer, and its overexpression is associated with poor prognosis. This may be due to the fact that ovarian cancer cells rely on CPT1A-mediated FAO to maintain growth and malignant phenotypes [20]. The expression of CPT1A was significantly upregulated in acute leukemia cells. Survival analysis demonstrated that the prognosis of patients with high expression of CPT1A was significantly worse than that of patients with low expression of CPT1A [21]. Subsequent studies confirmed that the small molecule inhibitor of CPT1A can reduce fatty acid oxidation in tumor cells of leukemia and exert an anticancer effect by inhibiting cell proliferation and inducing apoptosis [22]. The study also demonstrated that CPT1A was significantly correlated with tumor radiosensitivity. Nasopharyngeal carcinoma studies have confirmed that a reduction in the expression of CPT1A can improve the efficacy of radiotherapy for the disease [23]. Metastasis is the primary reason to which the difficulty in achieving a complete cure for cancer and subsequent treatment failures attributed. Recently, researchers observed that CPT1A expression was significantly upregulated in colorectal cancer. The upregulated CPT1A promoted metastasis in colorectal cancer by activating fatty acid oxidation, indicating that CPT1A is closely associated with tumor metastasis [24]. However, recent research seems to imply that CPT1A may play a different role in renal clear cell carcinoma compared to that in other tumors [16, 25].

This study was conducted to understand the potential role of this enzyme in KIRC. We assessed data from the KIRC dataset of the TCGA database and used the clinical data collected from our work for verification. The results indicate that the expression of CPT1A in kidney cancer tissue is significantly lower than that in normal kidney tissue. The survival curve also showed that CPT1A expression is associated with the overall survival of kidney cancer patients. In the KIRC dataset of the TCGA database, we observed that CPT1A expression is related to the tumor, metastasis, grade, and stage of patients with RCC. The clinicopathological data collected by us demonstrated that the expression of CPT1A is associated with the tumor size and Fuhrman classification of KIRC patients. To study the biological effects of CPT1A in vitro, we established 786-O and ACHN cell lines overexpressing CPT1A through plasmid transfection. CCK-8, wound healing, and Transwell invasion experiments were performed. The 786-O and ACHN cell lines overexpressing CPT1A demonstrated reduced proliferation, migration, and invasion capabilities.

We conducted a detailed bioinformatics analysis to further explore the biological role of CPT1A in KIRC. First, we used the String website to find ten genes associated with CPT1A and generated a PPI network. Subsequently, the R language was used to obtain the coexpression map of the eleven genes. CPT1A demonstrated a significant positive correlation with most genes. The heat map shows that the eleven genes exhibit varying degrees of disparity in KIRC. Hazard ratio analysis demonstrated that CPT1A and most of the associated genes act as protective factors in KIRC. Subsequently, a LASSO regression curve was used to establish a risk model associated with the prognosis of patients in KIRC. The risk signature included four genes, namely, CPT1A, LPL, CPT2, and EHHADH. In multivariate Cox analysis, age, grade, stage, and risk score were established as independent risk factors for KIRC. The final GSEA analysis demonstrated that CPT1A may exert biological effects in KIRC via a variety of potential biological pathways. CPT1A demonstrated potential as a novel prognostic indicator and potential therapeutic target for KIRC. We believe that this model can help clinicians make favorable treatment plans based on the prognosis prediction of the patient.

Previous studies examining the role of CPT1A in renal clear cells have observed that the HIF gene can directly target the CPT1A gene and subsequently suppress the development of the tumor by eliminating the deposited lipid [16]. In addition, the concept of “tumor slimming” has been proposed, demonstrating various ways to eliminate abnormally deposited lipids in renal clear cell carcinoma exerting a tumor-suppressive effect [26]. In recent years, researchers have discovered that both melatonin/PGC1A/UCP1 and PLCL1/UCP1 pathways promote KIRC “slimming” and eliminate abnormal lipid accumulation, thereby inhibiting the progress of KIRC [26, 27]. Tumor “slimming” is particularly important in renal clear cell carcinoma. We observed via GSEA analysis that CPT1A can stimulate the adipocytokine signaling pathway and citrate cycle/TCA cycle, which play an important role in eliminating abnormally accumulated lipids. Moreover, the oxidative phosphorylation pathway as a whole demonstrated an inhibitory effect, indicating that the overexpression of CPT1A does not produce a large amount of ATP in KIRC but instead plays a role in the inhibition of ATP synthesis. However, CPT1A in ovarian cancer, leukemia, and colorectal cancer primarily promotes the occurrence and development of tumors by promoting FAO, which can result in a large amount of ATP. Up to this point, we seem to be able to explain, to a certain extent, why CPT1A acts as a tumor suppressor gene in renal clear cell carcinoma, instead of acting as an oncogene in ovarian cancer, leukemia, and colorectal cancer. This requires further experimental verification.

## 5. Conclusions

Here, we observed, for the first time, that the low expression level of CPT1A in KIRC patients is associated with poor overall survival rate, larger tumor size, and higher tumor grade. In addition, the overexpression of CPT1A may reduce the proliferation, migration, and invasion of renal clear cell carcinoma cells in vitro. We used four genes, namely, CPT1A, LPL, CPT2, and EHHADH, to establish a prognostic risk signature for KIRC patients. GSEA analysis demonstrated that CPT1A may achieve a tumor-suppressing effect in KIRC via tumor “slimming.” However, there are certain limitations to this study. First, the association between CPT1A expression and KIRC biological behavior has not been confirmed in vivo. In addition, the potential molecular mechanism of CPT1A in the inhibition of renal clear cell carcinoma has not been elucidated completely. Further research is required to overcome these limitations. The results of this study indicate that CPT1A may be a promising prognostic and therapeutic biomarker for RCC.

## Figures and Tables

**Table 1:** Association between CPT1A expression in KIRC tissues and clinicopathological features and demographic data (*n* = 50).

Features	Number	CPT1A expression		*χ* ^2^	*P*
		High (*n* = 23)	Low (*n* = 27)		
Gender					
Male	37	19	18	1.641	0.200
Female	13	4	9		
Age					
≤50	14	7	7	0.125	0.723
>50	36	16	20		
Location side					
Left	23	11	12	0.057	0.811
Right	27	12	15		
Tumor size (cm)					
≤4.0	29	17	12	4.428	0.035^∗^
>4.0	21	6	15		
Fuhrman grade					
G1+G2	38	21	17	5.469	0.019^∗^
G3+G4	12	2	10		
TNM stage					
I+II	39	20	19	1.991	0.158
III+IV	11	3	8		

The analysis was performed using Fisher's exact test, and ∗ indicates statistical significance (*P* < 0.05).

## Data Availability

The data used to support the findings of this study are available from the corresponding author upon request.

## Abbreviations

RCC: Renal cell carcinoma
KIRC: Kidney renal clear cell carcinoma
TCGA: The Cancer Genome Atlas
CPT1A: Carnitine palmitoyltransferase 1A
LPL: Lipoprotein lipase
CPT2: Carnitine palmitoyltransferase 2
GSEA: Gene set enrichment analysis
EHHADH: Enoyl-CoA hydratase and 3-hydroxyacyl CoA dehydrogenase
CPT1B: Carnitine palmitoyltransferase 1B
CPT1C: Carnitine palmitoyltransferase 1C
VHL: Von Hippel-Lindau tumor suppressor
HIF: Hypoxia-inducible factor
CCK-8: Cell Counting Kit-8
PPI: Protein-protein interaction network
KIRP: Kidney renal papillary cell carcinoma
KICH: Kidney chromophobe
LASSO: Least absolute shrinkage and selection operator
PPARA: Peroxisome proliferator-activated receptor alpha
PPARGC1A: Peroxisome proliferator-activated receptor gamma coactivator 1 alpha
ACOX1: Acyl-CoA oxidase 1
FAO: Fatty acid oxidation
PGC1A: Peroxisome proliferator-activated receptor gamma
UCP1: Uncoupling protein 1
PLCL1: Phospholipase C like 1
ATP: Adenosine triphosphate.
